# A case of long QT syndrome type 2 that developed torsades de pointes two days after the initiation of oral β-blocker therapy

**DOI:** 10.1016/j.hrcr.2022.08.003

**Published:** 2022-08-09

**Authors:** Fumiya Yoneda, Takeru Makiyama, Kosuke Miyahara, Yoshitomo Fukuoka, Takeshi Aiba, Takeshi Kimura

**Affiliations:** ∗ Department of Cardiovascular Medicine, Kyoto University Graduate School of Medicine, Kyoto, Japan; † Department of Cardiovascular Medicine, Sugita Genpaku Memorial Obama Municipal Hospital, Obama, Japan; ‡ Department of Community Medicine Supporting System, Kyoto University Graduate School of Medicine, Kyoto, Japan; § Department of Cardiovascular Medicine, National Cerebral and Cardiovascular Center, Suita, Japan

**Keywords:** Long QT syndrome, β blocker, Torsades de pointes, Mexiletine, Sudden death

**Key Teaching Points**

• We experienced a case of long QT syndrome type 2 (LQT2) that developed ventricular tachyarrhythmias 2 days after the initiation of oral β-blocker therapy.
• During administration of β blockers in LQT2 patients, bradycardia and electrolyte abnormalities should be carefully monitored, since fatal arrhythmias can be provoked.
• Mexiletine can be a useful adjunctive medication to treat LQT2.

## Introduction

Congenital long QT syndrome (LQT) is a potentially lethal hereditary arrhythmic disorder that can cause syncope and sudden cardiac death owing to polymorphic ventricular tachycardias in association with prolonged QT intervals in electrocardiograms (ECGs), termed as “torsades de pointes” (TdP). The prevalence of LQT is reported to be 1 in 2000, and genetic testing reveals mutations in cardiac ion channel–related genes in about 70% of the cases. Variants in the 3 genes, *KCNQ1*, *KCNH2*, and *SCN5A*, account for approximately 90% of LQT cases, referred to as LQT type 1, 2, and 3 (LQT1, LQT2, and LQT3), respectively. Classification of these subtypes is important, since specific lifestyle guidance and treatments are recommended according to each genotype.^1^ Regarding pharmaceutical therapies for LQT, β blockers are the first-line treatment to prevent arrhythmias, specifically for LQT1 and LQT2.^2^ Here, we report a case of a patient with LQT2 who experienced TdP 2 days after the initiation of a β-blocker therapy and discuss the cautions upon starting β blockers.

## Case report

**Figure 1:**
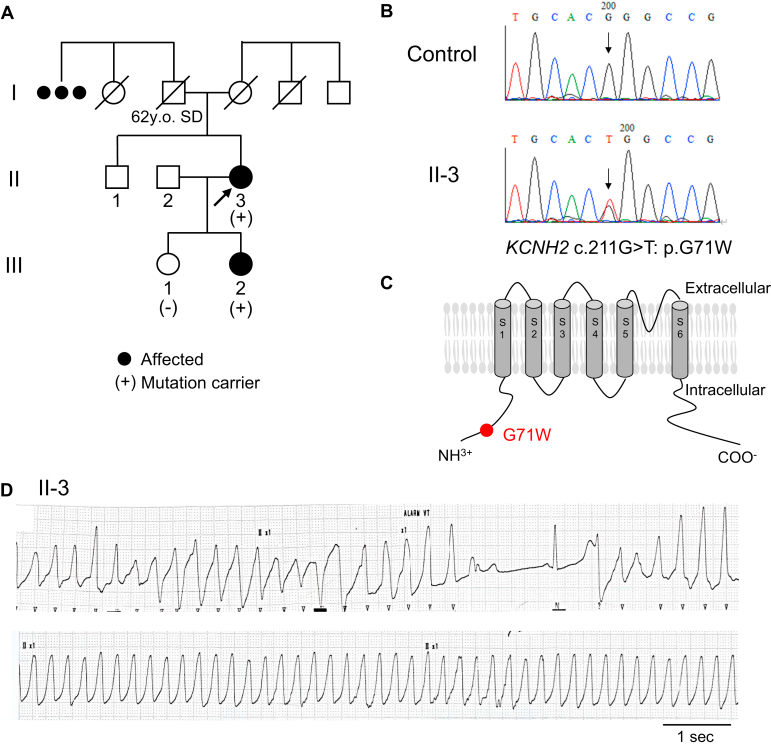
Pedigree, genotype, and a monitor electrocardiogram (ECG) of the proband (II-3). **A:** Family pedigree: arrow indicates the proband; filled circle, affected member; plus sign, *KCNH2* p.G71W carrier. SD = sudden death. **B:** DNA sequencing of the proband. **C:** Topology of the hERG channel and the location of the *KCNH2* variant, c.211G>T: p.G71W. **D:** ECG recording of the proband at the emergency department. Torsades de pointes was followed by a long-short sequence of R-R intervals.

A 41-year-old woman (Figure 1A; II-3, proband) had 2 syncopal episodes; the first one was at age 24 after waking up and the second was at age 37 after diarrhea, lasting for several days. During the second syncope, she exhibited hypokalemia (3.0 mEq/L) and transient marked QT interval prolongation on her ECG (heart rate [HR]: 69 beats per minute [bpm]; QTc: 692 ms). She was diagnosed with secondary LQT due to hypokalemia at that time. Potassium supplements were prescribed, and no further examination was conducted. In addition, she had been taking ethyl loflazepate to treat her depression. When she was 42 years old, her second daughter, at the age of 14 (Figure 1A; III-2), was diagnosed with LQT after a syncope, and genetic testing for 60 candidate genes for LQT using a benchtop next-generation sequencer (MiSeq; Illumina, San Diego, CA) was performed and the identified variant was confirmed by Sanger sequencing. A heterozygous missense variant, *KCNH2* c.211G>T: p.G71W (dbSNP: rs199473420; Figure 1B), was identified in the index patient (II-3) and her second daughter (III-2), but not in the unaffected first daughter (III-1; Figure 1A). The variant, located in the N-terminus of the hERG channel (Figure 1C), was previously reported in LQT cases.^3^ Though the classification of this variant differs depending on the database, pathogenic in the VarSome and of uncertain significance in the ClinVar (ClinVar Variation ID: 853484), the proband and her second daughter were diagnosed with LQT2. As an initial treatment, the index patient (II-3) started taking nadolol (0.45 mg/kg/day; her body weight: 67 kg) and was followed up at an outpatient clinic. However, 2 days later, she was transferred to the emergency room owing to general malaise and decreased level of consciousness. The telemetry ECG showed TdP followed by a long-short sequence of R-R intervals and sustained ventricular tachycardia (VT) (Figure 1D). TdP was initiated by a premature ventricular contraction with a long coupling interval (640 ms), which is a typical feature observed in LQT-associated TdPs. Since she was stable during VT, a bolus dose (50 mg) of lidocaine was administered, which successfully converted the VT to normal sinus rhythm in approximately 2 minutes.

**Figure 2:**
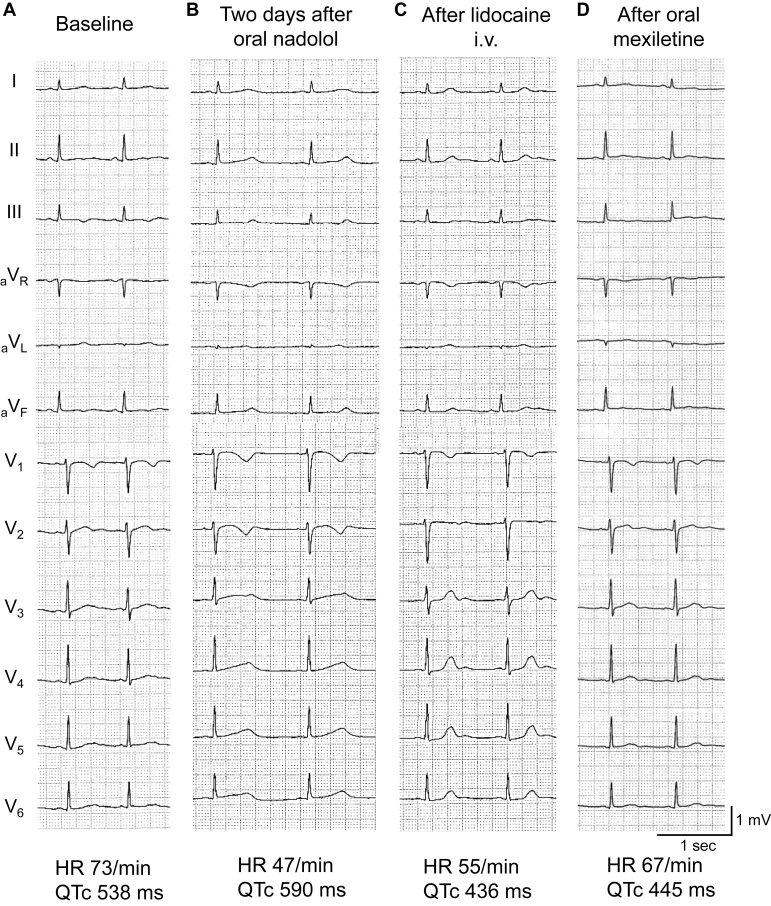
Electrocardiograms (ECGs) of the proband (II-3). ECGs recorded **A:** before the initiation of nadolol, **B:** 2 days after the initiation of nadolol (0.45 mg/kg/d), **C:** immediately after intravenous administration of lidocaine (50 mg) at the emergency room, and **D:** under oral mexiletine (4.5 mg/kg/d). QTc values were calculated using Bazett’s formula.

Figure 2 shows 12-lead ECGs of the index patient (II-3) at multiple events. Before starting nadolol, her HR was 73 bpm and QTc was 538 ms (Figure 2A). The ECG recorded 2 days after nadolol showed bradycardia with HR of 47 bpm and a QTc of 590 ms (Figure 2B). The ECG after the injection of lidocaine at the emergency room showed a remarkably shortened QTc of 436 ms (Figure 2C). She was observed at an intensive care unit with a continuous infusion of lidocaine (0.9 mg/kg/h). Her echocardiography showed preserved left ventricular contractility and no abnormal findings were noted. The electrolytes on admission were Na 136 mEq/L (normal range: 135–147 mEq/L), K 3.7 mEq/L (3.3–4.8 mEq/L), Ca 9.4 mg/L (8.7–11.0 mg/dL), and Mg 1.6 mg/dL (1.8–2.6 mg/dL), indicating a mildly lower level of potassium and the magnesium concentration below the normal range. As the bradycardia was considered as a cause of TdP, nadolol was discontinued. On the seventh day, the continuous lidocaine infusion was switched to oral mexiletine hydrochloride (4.5 mg/kg/d). In addition, oral potassium gluconate (0.24 mEq/kg/d) and spironolactone (0.19 mg/kg/d) were added to correct hypokalemia. The dose of potassium gluconate was increased to 0.45 mEq/kg/d and the patient was discharged on day 21. An implantable cardioverter-defibrillator (ICD) implantation was recommended; however, she refused it. She has been on mexiletine hydrochloride (4.5 mg/kg/d) and potassium gluconate (0.24 mEq/kg/d) for 1 year without any events. Her ECG parameters remain within near-normal ranges: HR 60–70 bpm and QTc 445–470 ms (Figure 2D).

**Figure 3:**
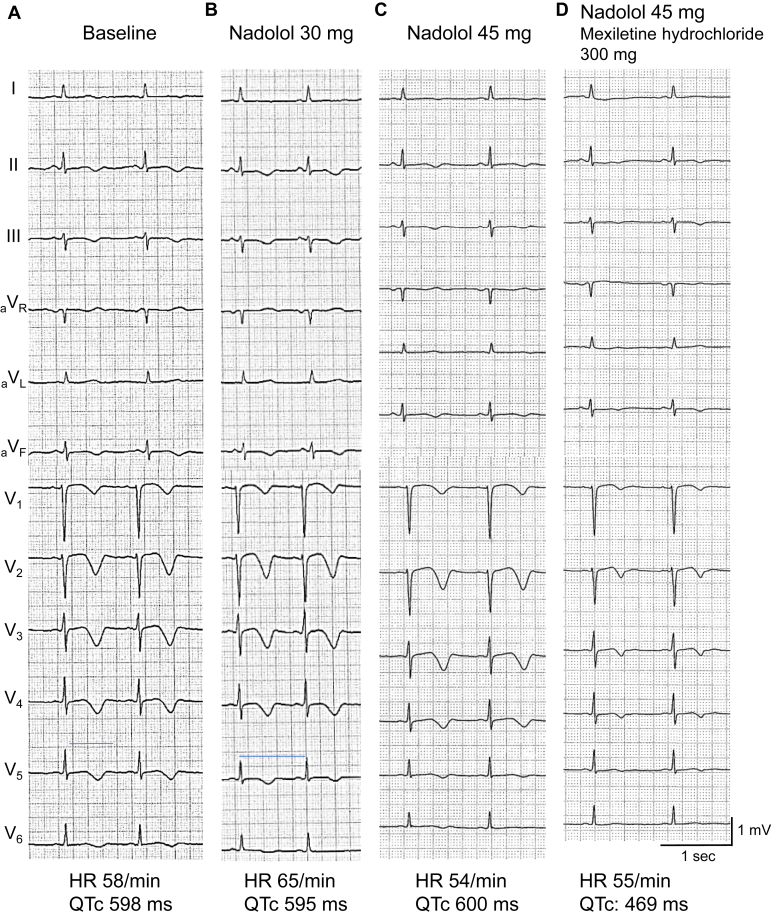
Electrocardiograms (ECGs) of the second daughter (III-2). ECGs recorded **A:** at baseline, **B:** after the initiation of nadolol (0.64 mg/kg/d), **C:** after dose increase of nadolol (0.96 mg/kg/d), and **D:** after the addition of mexiletine (6.4 mg/kg/d). QTc values were calculated using Bazett’s formula.

Her second daughter (Figure 1A; III-2), who had 3 syncopal episodes, was also treated with an oral administration of nadolol (0.64 mg/kg/d, her body weight: 47 kg) after the genetic diagnosis of LQT2. Figure 3 shows her ECGs with different medications. Her QTc intervals before and after nadolol were similar (Figure 3A and 3B). Four months later, she had another syncope just after running. The dose of nadolol was increased to 0.96 mg/kg/d which is within the therapeutic target range (0.75–2 mg/kg/d); however, the QTc interval remained long (600 ms) (Figure 3C). During her observation, she also started taking oral mexiletine because her mother (II-3) had TdP associated with nadolol. She has been following up with nadolol (0.96 mg/kg/d) and mexiletine hydrochloride (6.4 mg/kg/d) for 1 year without any events. Her average HR is currently around 55–60 bpm and QTc interval is mildly shortened to a range of 465–530 ms (Figure 3D).

## Discussion

In LQT patients, β blockers are the first line of therapy.^2^ Moss and colleagues^4^ reported that β blockers reduced cardiac events, including unexplained syncope, by 85% in LQT1 and by 65% in LQT2. Compared to other β blockers, 2 β blockers for LQT patients, propranolol and nadolol, have multiple evidences for drug efficacy; specifically, Abu-Zeitone and colleagues^5^ reported that only nadolol provided significant risk reduction for first cardiac events (hazard ratio: 0.40 [95% confidence interval 0.16–0.98]).

In this case report, a patient with LQT2 who had a history of recurrent syncope was treated with nadolol. However, lethal ventricular arrhythmias occurred just 2 days after the initiation of the therapy. To the best of our knowledge, this is the first report of TdP occurring immediately after the initiation of β blockers in patients with LQT. The patient was considered to be high-risk for a fatal arrhythmic event owing to a history of syncope and a markedly prolonged QT interval (QTc: 538 ms at baseline [>500 ms]).^2^ According to the recent report by Mazzanti and colleagues,^6^ LQT2 patients with a QTc range of 531–540 ms may have 7.6 life-threatening arrhythmia events within 5 years. Moving forward, the question is “Why did this patient develop TdP?” We can speculate a couple of possible mechanisms. First, bradycardia, worsened by nadolol, may have contributed to the development of TdP. The patient was started on approximately half the target dose of nadolol (0.45 m/kg/d) in order to take precautions against sudden excessive bradycardia and drug intolerance. However, within 2 days, she developed bradycardia with HR of 48 bpm, which resulted in TdP associated wtih QT prolongation. Secondly, the patient showed a mildly lower level of potassium and hypomagnesemia, which might have facilitated the TdP. Roden^7^ reported that bradycardia in hypokalemic Purkinje fibers using rapid inward rectifier potassium current (I_Kr_) blocking drugs prolonged the action potential durations of M cells, resulting in an increase in transmural dispersion of refractoriness and QT prolongation. Thus, nadolol-induced bradycardia and mild electrolyte abnormalities might have caused the QT prolongation, resulting in TdP in the index patient.

In the index patient, mexiletine, a class Ib antiarrhythmic agent, shortened the QTc intervals (Figure 2A and 2D). In her second daughter, when mexiletine was added to nadolol (0.96 mg/kg/d) by oral administration, further improvement in QTc prolongation was observed (Figure 3C and 3D). Thus far, there are several reports that class Ib antiarrhythmic agents are effective in the treatment of patients with LQT2. Clinically, mexiletine significantly shortened QTc intervals by an average of 65 ms in 12 patients with LQT2.^8^ Experimentally, in canine left ventricular wedge preparations, in which LQT2 was reproduced by I_Kr_ inhibition with d-sotalol, mexiletine treatment reduced the transmural dispersion of repolarization and suppressed TdP.^9^ As for the mechanism by which class Ib antiarrhythmic agents shorten prolonged QT intervals in LQT2, late Na^+^ current (I_NaL_) blockade has been considered. I_NaL_ have important roles in the arrhythmogenic pathogenesis of LQT2: (1) enhanced I_NaL_ provide additional depolarizing currents during action potential plateau phase, and (2) increased intracellular Na^+^ reduces the depolarizing Na^+^/Ca^2+^ exchanger, resulting in the suppression of the membrane potential during action potential plateau phase and delaying the activation of slow components of delayed rectifier K^+^ current (I_Ks_).^10^ Thus, inhibition of I_NaL_ is one of the effective treatments for LQT2 cases, as well as drug-induced LQT, which is mainly caused by I_Kr_ inhibition.^11^

In the treatment of LQT2 patients, potassium supplementation and mexiletine can be useful pharmacological adjunctive therapies, as mentioned. For those who are resistant to oral therapies, nonpharmacological treatments, such as ICDs, pacing, and left cardiac sympathetic denervation (LCSD) must be considered.^2^ In LQT2, where the vast majority of lethal arrhythmias are pause/bradycardia dependent,^12^ atrial pacing has been considered to be a useful adjunctive therapy. Recently, Kowlgi and colleagues^13^ reported that intentional permanent atrial pacing for LQT2 significantly shortened QTc intervals and reduced cardiac event risk from 1.01 to 0.02/patient-year. ICD implantation will reduce the mortality rate of LQT patients by preventing TdP through avoiding bradycardia via atrial pacing and electrical defibrillation of lethal arrhythmias.^14^ ICD implantation is considered for LQT patients who are survivors of a cardiac arrest (class I) and for patients who experience recurrent syncopal events while on β-blocker therapy (class IIa).^2^ However, indications of cardiac implantable electric devices must be carefully determined because device implantation in young patients involves the risk of complications, such as infection from frequent battery replacement. LCSD is considered for patients on β blockers and with uncontrolled fatal arrhythmias after ICD implantation.^2^ Very recently, Dusi and colleagues^15^ reported that the protective effect of LCSD is not influenced by common genotypes. LCSD significantly reduced the mean yearly rate of cardiac events by 95% in 18 LQT1 patients (from 1.14 to 0.08/patient-year) and by 86% in 27 LQT2 patients (from 1.42 to 0.34/patient-year), respectively.

When starting β blockers in LQT patients, the risk of cardiac events due to bradycardia-induced TdP might be taken into consideration, as shown in the present case, although it is considered rare. Electrolyte abnormalities should be corrected immediately; and in the high-risk cases of LQT, initiation of β blockers during hospitalization or combination with wearable cardioverter defibrillators may be useful. But it is difficult to perform these treatments in all cases and the stratification of high-risk patients with LQT at β-blocker introduction is an important issue to be elucidated in the future.

## Conclusion

Although β-blocker therapy is considered as the first-line therapy in treating LQT, this is the first report of an LQT2 case in which the patient presented TdP shortly after starting nadolol. Bradycardia induced by the β blocker and even subtle electrolyte abnormalities can be associated with TdP. Close monitoring of heart rate and QT intervals should be considered when β-blocker therapies are initiated in LQT patients.
